# Multicompartmental prolapse: A comparative study between clinical examination and ultrasound

**DOI:** 10.1002/ijgo.70886

**Published:** 2026-02-21

**Authors:** José Antonio García‐Mejido, Olaya Salas‐Alvarez, Fernando Bugatto‐Gonzalez, Ana Fernández‐Palacín, Fernando Fernández‐Palacín, José Antonio Sainz‐Bueno

**Affiliations:** ^1^ Department of Surgery, Faculty of Medicine University of Seville Seville Spain; ^2^ Institute of Biomedicine of Seville (IBiS) Seville Spain; ^3^ Department of Child and Mother Health and Radiology, School of Medicine University of Cádiz Cádiz Spain; ^4^ Division of Maternal‐Fetal Medicine, Obstetrics and Gynecology Department Puerta Del Mar University Hospital Cádiz Spain; ^5^ Biomedical Research and Innovation Institute of Cádiz (INiBICA) Cádiz Spain; ^6^ Biostatistics Unit, Department of Preventive Medicine and Public Health University of Seville Seville Spain; ^7^ Department of Statistics and Operational Research University of Cádiz Cádiz Spain

**Keywords:** pelvic organ prolapse, POP‐Q system, surgery, ultrasonography

## Abstract

**Objective:**

The accurate diagnosis of multicompartment pelvic organ prolapse (POP) is fundamental to surgical success. This study aims to compare the diagnostic performance of transperineal ultrasound against clinical examination (pelvic organ prolapse quantification, POP‐Q) for the detection of compartmental defects in patients with multicompartment POP, using assessment under spinal anesthesia as the reference standard.

**Method:**

A prospective randomized diagnostic accuracy study was designed, including 129 patients scheduled for multicompartment POP surgery. Patients were randomly assigned to undergo either a preoperative POP‐Q two‐dimensional transperineal ultrasound assessment. The reference standard for all patients was the intraoperative POP‐Q assessment, conducted under spinal anesthesia immediately before surgery. Sensitivity, specificity, and likelihood ratios (LR) were calculated for each method and compartment.

**Results:**

Both techniques demonstrated high sensitivity for the diagnosis of cystocele (100% vs 98.3%). However, their performance varied across the other compartments. Ultrasound showed superior specificity for uterine prolapse (73.4% vs 45.4%) and rectocele (86.3% vs 66.0%) and was particularly robust in confirming enterocele (LR+ of 10.5). In turn, clinical examination had a higher sensitivity for detecting rectocele (61.5% vs 21.4%) and was highly reliable for ruling out cystocele and uterine prolapse (LR− of 0).

**Conclusion:**

Clinical examination and ultrasound are complementary in the diagnosis of prolapse. Their combined use is key to accurate surgical planning.

**Abbreviations**
BMI: body mass index
LR: likelihood ratios
POP: pelvic organ prolapse
POP‐Q: pelvic organ prolapse quantification

## 1 INTRODUCTION

Pelvic organ prolapse (POP), defined as the descent of one or more of these organs through the vaginal canal,^1^, ^2^, ^3^, ^4^, ^5^, ^6^ is a highly prevalent condition with a significant impact on women's health. Frequently, POP is not limited to a single defect but manifests as a multicompartment entity, in which the anterior, central, and/or posterior compartments are simultaneously involved. This anatomical reality adds a layer of complexity to both accurate diagnosis and the subsequent surgical approach.^3^ In this context, obtaining a highly accurate anatomical diagnosis is an indispensable prerequisite for outlining a successful therapeutic plan aimed at minimizing long‐term recurrence rates.^7^

Standardized clinical evaluation, articulated through the Pelvic Organ Prolapse Quantification (POP‐Q) system, constitutes the diagnostic cornerstone of routine clinical practice.^2^, ^3^, ^6^, ^8^ However, it is well known that its reliability in the office setting can be limited by several factors, such as the inherent variability in the patient's performance of the Valsalva maneuver, the potential influence of bladder or rectal filling status, or the co‐activation of the levator ani musculature.^1^, ^5^, ^9^, ^10^, ^11^, ^12^, ^13^, ^14^

In contrast, the assessment of prolapse under anesthesia, either general or spinal, offers a qualitatively different diagnostic scenario.^5^, ^10^, ^15^, ^16^, ^17^ By inducing complete relaxation of the pelvic musculature and eliminating factors that can inhibit an effective strain, a passive and often more pronounced exteriorization of the prolapsed compartments is facilitated.^5^, ^9^, ^10^, ^15^, ^16^, ^17^, ^18^, ^19^ This confluence of factors has led the scientific community to suggest that intraoperative assessment might provide a more complete and faithful representation of the anatomical defect, thus constituting an evaluation that more closely approximates the true structural reality of the prolapse.^5^, ^9^, ^10^, ^15^, ^16^, ^18^, ^19^

Separately, transperineal pelvic floor ultrasound has strongly emerged as a complementary and objective diagnostic tool.^1^, ^2^, ^4^, ^19^, ^20^, ^21^ This imaging modality enables a dynamic assessment of pelvic floor anatomy and function, allowing not only for the reproducible quantification of the descent of different organs but also for the morphological evaluation of the main support structures.^1^, ^4^, ^13^, ^20^, ^22^, ^23^ This technique's ability to provide objective anatomical and functional data could, therefore, significantly complement the more dimensional information offered by clinical examination.^1^, ^2^, ^4^, ^19^, ^21^

However, a fundamental limitation exists in the current scientific evidence, as most studies comparing ultrasound with clinical examination have used the office‐based examination as the reference, a standard whose ability to reveal the maximum extent of the prolapse has been questioned.^1^, ^5^, ^9^, ^10^, ^11^, ^15^, ^16^, ^18^ For this reason, a knowledge gap remains regarding how ultrasound findings correlate with the true magnitude of the prolapse observed under the optimal conditions for exteriorization afforded by the surgical setting.^1^, ^4^, ^19^, ^24^, ^25^

The main objective of this study is, therefore, to compare the diagnostic performance of transperineal pelvic floor ultrasound versus clinical examination with the POP‐Q system for the detection of compartmental defects in patients with multicompartment prolapse. To this end, intraoperative clinical assessment under spinal anesthesia will be used as the reference standard most representative of the true anatomical defect.

## 2 MATERIALS AND METHODS

This was a prospective randomized diagnostic accuracy study conducted at the Gynecology and Obstetrics Unit of the Valme University Hospital in Seville between January 2022 and December 2023. The study adhered to the STARD (Standards for Reporting Diagnostic Accuracy Studies) guidelines.

The reference standard for all participants was the intraoperative POP‐Q assessment performed under spinal anesthesia immediately prior to the surgical repair.

The study included patients with an indication for surgical treatment due to symptomatic multicompartment POP. This condition was operationally defined as clinical evidence of descent to Stage II or higher, according to the POP‐Q system, in at least two of the three pelvic compartments (anterior, central, or posterior),^2^ and where such descent was symptomatic for the patient. The sole exclusion criterion was a history of previous corrective surgery for POP or any other pelvic floor surgical intervention.

**FIGURE 1:**
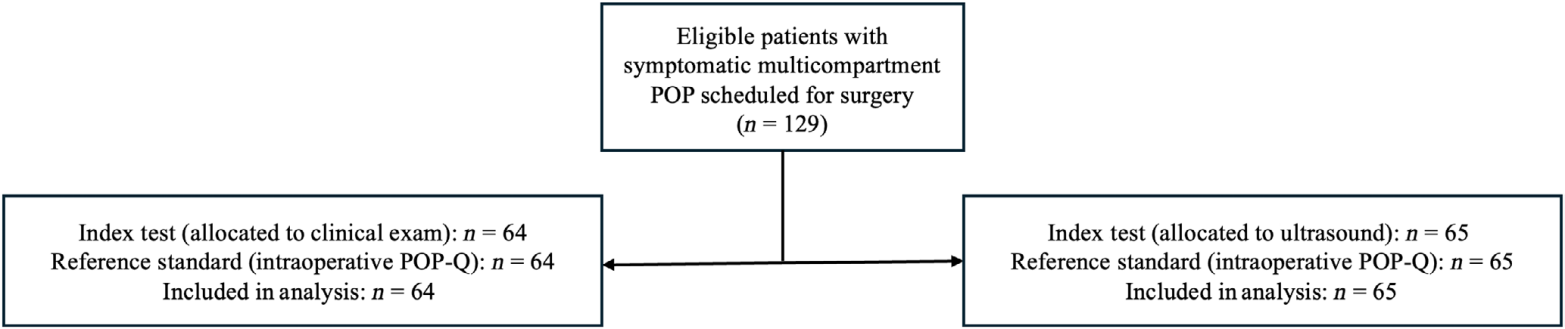
STARD (Standards for Reporting Diagnostic Accuracy Studies) flow diagram. Flow of participants through the study. A total of 129 eligible patients with symptomatic pelvic organ prolapse were randomized to either the preoperative clinical examination group (*n* = 64) or the preoperative ultrasound group (*n* = 65). All participants underwent the assigned index test followed by the intraoperative reference standard, with no missing data or withdrawals.

Participant recruitment was carried out using consecutive sampling. After inclusion, participants were randomly assigned to one of two preoperative assessment arms: (i) clinical evaluation using the POP‐Q system, or (ii) two‐dimensional transperineal ultrasound (Figure 1).

### 2.1 Preoperative clinical evaluation

The clinical evaluation was performed by a single specialist gynecologist with over 10 years of experience in pelvic floor disorders, who was unaware of the patient's initial clinical examination findings. This assessment was standardized through a systematic physical examination with the patient in the dorsal lithotomy position, using the POP‐Q system.^5^, ^6^, ^8^, ^10^

### 2.2 Preoperative ultrasound evaluation

Patients assigned to this arm underwent a two‐dimensional transperineal ultrasound assessment. Examinations were performed using a Toshiba 700 Aplio ultrasound system, equipped with a convex volumetric probe (PVT‐675 MV). The assessments were conducted by a gynecologist with over 10 years of experience in pelvic floor imaging, who was blinded to the clinical examination findings.

The procedure was standardized with the patient in the lithotomy position after ensuring complete bladder emptying.^1^, ^12^, ^19^, ^25^ During the examination, video sequences (cine‐loops) were recorded in the midsagittal plane, starting with the patient at rest and continuing through a maximal and sustained Valsalva maneuver (for a minimum of 6 s).

**FIGURE 2:**
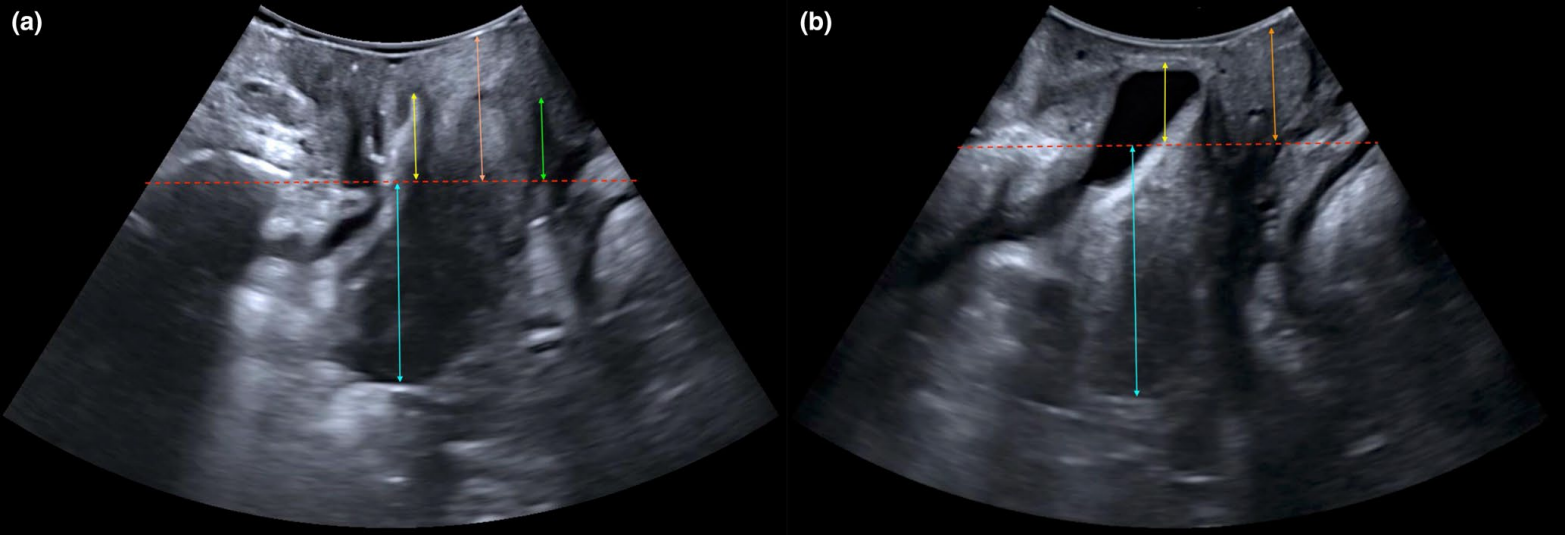
Two multicompartment prolapses. (a) Cystocele, uterine prolapse, and rectocele. (b) Cystocele and cervical elongation. Dashed red line: Posteroinferior pubic border. Yellow arrow: Bladder descent. Orange arrow: Cervical descent. Light blue arrow: Pubo‐uterine fundus distances. Green arrow: Herniation of the rectal ampulla.

Measurements for the diagnosis of the different compartmental defects were performed offline, analyzing these saved videos and using the posteroinferior margin of the pubic symphysis as the anatomical reference line. The following diagnostic criteria were defined: cystocele (bladder descent ≥10 mm during Valsalva)^21^ (Figure 2), uterine prolapse (a difference of ≥15 mm between the pubo‐uterine fundus distances at rest and on Valsalva, with a cervical descent of ≥15 mm)^19^ (Figure 2), cervical elongation (a difference of <15 mm between the pubo‐uterine fundus distances at rest and on Valsalva but with a cervical descent of ≥15 mm during Valsalva relative to the posteroinferior margin of the pubic symphysis)^19^ (Figure 2), rectocele (herniation of the rectal ampulla ≥15 mm on Valsalva)^21^, ^26^ (Figure 2), and enterocele (descent of peritoneal contents ≥15 mm on Valsalva).^26^

### 2.3 Reference standard: Intraoperative clinical evaluation

The definitive diagnosis, which was considered the reference standard in our study, was established through a clinical evaluation performed in the operating room with the patient under spinal anesthesia.^5^, ^9^, ^10^, ^15^, ^16^, ^17^, ^19^ This examination was carried out by the lead surgeon (a gynecologist specializing in pelvic floor disorders), who remained blinded at all times to the results of the preoperative assessments (both clinical and ultrasound), again using the POP‐Q system to determine the presence and true extent of the prolapse in each compartment.^6^, ^8^ Surgical prolapse was defined as the objectification of a Stage II or greater descent under these optimal examination conditions.

### 2.4 Postoperative clinical evaluation

The postoperative evaluation, like the preoperative examination, was conducted in the outpatient setting by the same pelvic floor specialist, who again used the POP‐Q system to ensure consistency in measurements.^6^

### 2.5 Statistical analysis

For this study, all statistical analyses were performed using SPSS software for Windows, version 25.0 (IBM, Armonk, NY, USA). Quantitative variables, expressed as mean and standard deviation, were compared between study groups using the independent samples *t*‐test. For the analysis of qualitative variables, presented as frequencies and percentages, the *χ* ^2^‐test or, when application conditions required, Fisher's exact test, was used.

Further, the ability of both clinical examination and ultrasound to predict surgical findings was assessed. This was conducted through a diagnostic performance analysis, calculating sensitivity, specificity, and likelihood ratios (LR) (positive and negative), along with their corresponding 95% confidence intervals.

To determine the required sample size, the ability to detect a difference of at least 25% in diagnostic accuracy between clinical examination and transperineal ultrasound was assumed. These values were based on the concordance with intraoperative findings for anterior compartment prolapse as previously reported in the literature.^19^ Based on these premises, it was calculated that a sample size of 120 patients would be sufficient to achieve an *α* error of 5% and a statistical power of 85%. This sample size calculation was performed using G*Power software, version 3.1.

### 2.6 Ethical approval

The study protocol underwent rigorous evaluation and received explicit approval from the Clinical Research Ethics Committee of the Ntra. Sra. de Valme University Hospital (approval code: 1259‐N‐20). In strict accordance with the ethical principles of the Declaration of Helsinki, all participants were thoroughly informed about the study's objectives and procedures and provided written informed consent before their inclusion.

## 3 RESULTS

A total of 129 patients were included in the study, 64 of whom did not have a preoperative ultrasound assessment and 65 did. Both patient groups were comparable in most of their baseline characteristics, such as age at menopause, body mass index (BMI, calculated as weight in kilograms divided by the square of height in meters), and the number of cesarean sections and abortions. However, statistically significant differences were found in the number of deliveries, with a mean of 2.9 deliveries in the group without ultrasound compared to 2.4 in the group that did (*P* = 0.030).

The prevalence of certain prolapses varied notably between the groups, as detailed in Table 1. Uterine prolapse was significantly more common in the group without ultrasound (78.1%) than in the group with ultrasound (32.3%) (*P* < 0.001). Similarly, cervical elongation was considerably more frequent in the group with ultrasound (58.5%) compared to 9.4% in the group without ultrasound (*P* < 0.001).

**TABLE 1:** General preoperative characteristics of patients in the clinical and ultrasound evaluation groups.

	Preoperative clinical evaluation (*n* = 64)	Preoperative ultrasound evaluation (*n* = 65)	*P*	95% CI
Age at menopause (years)	50.7 ± 3.5	50.2 ± 4.2	0.721	−1; 2
BMI	27.2 ± 4.3	27.8 ± 4.8	0.226	−2.2; 9.7
Obstetric history				
Parity	2.9 ± 1.3	2.4 ± 1.1	0.030	0.001; 1
Cesarean sections	0.05 ± 0.2	0.1 ± 0.3	0.198	−0.001; 0.001
Abortions	0.3 ± 0.7	0.3 ± 0.7	0.741	−0.001; 0.001
Presence of cystocele	62 (96.9)	59 (90.8)	0.270	3.0; 14.8
Stage I	2 (3.2)	2 (3.4)	0.310	−7.7; 7.3
Stage II	7 (11.3)	13 (22.0)		−23.8; 2.9
Stage III	53 (85.5)	44 (74.6)		3.6; 24.8
Presence of uterine prolapse	50 (78.1)	21 (32.3)	<0.001	29.3; 59.6
Stage I	2 (4)	2 (4.8)	0.008	−16.1; 10.2
Stage II	5 (10)	8 (38.1)		−49.3; −5.8
Stage III	32 (64)	12 (57.1)		−17.2; 31.1
Stage IV	11 (22)	0 (0)		4.6; 32.9
Presence of cervical elongation	6 (9.4)	38 (58.5)	<0.001	−61.6; −33.7
Stage I	0 (0)	2 (5.3)	0.042	−19.3; 29.3
Stage II	1 (16.7)	22 (57.9)		−66.2; 1.2
Stage III	5 (83.3)	14 (36.8)		4.0; 71.0
Presence of rectocele	26 (40.6)	25 (38.5)	0.802	−14.5; 18.7
Stage I	10 (38.5)	12 (48.0)	0.391	−35.0; 17.3
Stage II	12 (46.2)	7 (28.0)		−8.5; 42.1
Stage III	4 (15.4)	6 (24.0)		−29.9; 13.7
Presence of enterocele	2 (3.1)	1 (1.5)	0.619	−4.9; 8.0
Stage I	0 (0)	0 (0)	1	−76.5; 59.8
Stage II	1 (50.0)	1 (100)		−89.1; 55.8
Stage III	1 (50.0)	0 (0)		−55.8; 89.1

*Note*: Values are presented as mean ± standard deviation or *n* (%).

Abbreviations: BMI, body mass index; CI, confidence interval.

In the postoperative assessment, presented in Table 2, no significant differences were observed between the groups regarding examination time or the prevalence of cystocele, uterine prolapse, cervical elongation, rectocele, or enterocele.

**TABLE 2:** Postoperative follow‐up assessment of patients in the clinical and ultrasound evaluation groups.

	Preoperative clinical evaluation (*n* = 64)	Preoperative ultrasound evaluation (*n* = 65)	*P*	95% CI
Follow‐up time (days)	87.1 ± 24.0	87.0 ± 23.4	0.843	−4.0; 4.0
Presence of cystocele	15 (23.4)	15 (23.1)	1	−14.2; 14.9
Stage I	12 (80.0)	11 (73.3)	0.651	−23.7; 35.5
Stage II	2 (13.3)	4 (26.7)		−40.0; 16.5
Stage III	1 (6.7)	0 (0)		−13.1; 24.8
Presence of uterine prolapse	0 (0)	1 (1.5)	1	−6.5; 3.6
Stage I	0 (0)	1 (100)	—	—
Stage II	0 (0)	0 (0)		—
Stage III	0 (0)	0 (0)		—
Stage IV	0 (0)	0 (0)		—
Presence of cervical elongation	0 (0)	1 (1.5)	1	—
Stage I	0 (0)	1 (100)	—	—
Stage II	0 (0)	0 (0)		—
Stage III	0 (0)	0 (0)		—
Presence of rectocele	12 (18.8)	14 (21.5)	0.827	−16.5; 11.2
Stage I	10 (83.3)	9 (64.3)		−15.9; 48.1
Stage II	2 (16.7)	5 (35.7)		−48.1; 15.9
Stage III	0 (0)	0 (0)		—
Presence of enterocele	0 (0)	2 (3.1)	0.496	—
Stage I	0 (0)	0 (0)	—	—
Stage II	0 (0)	2 (100)		—
Stage III	0 (0)	0 (0)		—

Abbreviation: CI, confidence interval.

The diagnostic ability to predict surgical POP of each organ in multicompartment prolapse was variable between clinical examination and ultrasound, as shown in Table 3. For cystocele, clinical examination and ultrasound had high sensitivity (100% and 98.3%, respectively). Clinical examination of uterine prolapse showed lower specificity than transperineal ultrasound (45.4% vs 73.4%). For cervical elongation, clinical examination showed higher specificity than ultrasound for diagnosing surgical prolapse (65.6% vs 40.6%). Clinical examination of rectocele had a higher sensitivity (61.5%) compared to ultrasound (21.4%). However, ultrasound showed greater specificity (86.3%) compared to clinical examination (66.0%). The specificity of ultrasound for enterocele (95.2%) was similar to clinical examination (97.6%), being high for both assessment methods.

**TABLE 3:** Diagnostic accuracy of clinical evaluation versus ultrasound in predicting surgical findings of POP (grade ≥2).

Type of prolapse	Clinical evaluation to predict surgical diagnosis		Ultrasound to predict surgical diagnosis	
	Sensitivity (95% CI)	Specificity (95% CI)	Sensitivity (95% CI)	Specificity (95% CI)
Cystocele	100 (96.9; 100)	0 (0; 60.2)	98.3 (90.6; 99.9)	0 (0; 84.2)
Uterine prolapse	100 (2.5; 100)	45.3 (36.5; 54.4)	0 (0; 97.5)	73.4 (60.9; 83.7)
Cervical elongation	0 (0; 97.5)	65.6 (56.7; 73.8)	100 (2.5; 100)	40.6 (28.5; 53.6)
Rectocele	61.5 (40.6; 79.8)	66.0 (56.0; 75.0)	21.4 (4.7; 50.8)	86.3 (73.7; 94.3)
Enterocele	0 (0; 84.2)	97.6 (93.3; 99.5)	50.0 (1.23; 98.7)	95.2 (86.7; 99.0)

Abbreviation: POP, pelvic organ prolapse.

The diagnostic performance, evaluated by LR, is presented in Table 4. In the analysis of diagnostic performance, clinical examination proved to be a robust tool to increase the probability of detecting a uterine prolapse and a rectocele (LR+ of 1.8 in both cases), and its negative result was especially powerful in ruling out the presence of cystocele and uterine prolapse (LR− of 0). For its part, ultrasound stood out for its high capacity to confirm the existence of an enterocele (LR+ of 10.5) and cervical elongation (LR+ of 1.7), while also being very reliable in ruling out the latter condition with a negative result (LR− of 0). However, for the diagnosis of cystocele by ultrasound, an anomalous result was observed, being a statistical artifact derived from a null specificity (0%) that invalidates the usefulness of a negative result to rule out the pathology.

**TABLE 4:** Diagnostic performance of clinical evaluation versus ultrasound using likelihood ratios.

Type of prolapse	Diagnostic performance of clinical evaluation		Diagnostic performance of ultrasound	
	Positive LR (LR+)	Negative LR (LR−)	Positive LR (LR+)	Negative LR (LR−)
Cystocele	1 (1; 1)	0 (0; 0)	0.98 (0.95; 1.02)	1.8 × 10^38^ (0; 1.8 × 10^38^)
Uterine prolapse	1.8 (1.6; 2.2)	0 (0; 0)	0 (0; 0)	1.4 (1.2; 1.6)
Cervical elongation	0 (0; 0)	1.5 (1.3; 1.7)	1.7 (1.4; 2.1)	0 (0; 0)
Rectocele	1.8 (1.2; 2.7)	0.6 (0.4; 1)	1.6 (0.4; 5.3)	0.9 (0.7; 1.2)
Enterocele	0 (0; 0)	1.0 (1; 1.1)	10.5 (1.8; 6.2)	0.5 (0.1; 2.1)

## 4 DISCUSSION

This prospective study aimed to compare the diagnostic performance of standardized presurgical clinical examination using the POP‐Q system against presurgical transperineal ultrasound, with intraoperative assessment under spinal anesthesia serving as the reference standard in patients with multicompartment POP. Our results indicate that neither diagnostic modality can be categorically considered superior to the other; in contrast, their performance varies significantly depending on the pelvic compartment being evaluated. Clinical examination, for instance, demonstrated a sensitivity of 100% for the diagnosis of uterine prolapse, markedly outperforming ultrasound in this regard. Conversely, transperineal ultrasound proved superior for identifying cervical elongation, with a sensitivity of 100%, and for detecting enterocele, where it yielded a positive likelihood ratio (LR+) of 10.5, a value indicative of an excellent ability to confirm the presence of this condition. These findings strongly suggest that clinical examination and ultrasound are, in fact, complementary tools, each with its own strengths and weaknesses within the complex diagnostic landscape of multicompartment prolapse.

The fundamental premise of our study is based on the growing body of evidence that prolapse assessment in the outpatient setting might underestimate the true extent of the anatomical defect.^10^, ^11^, ^15^, ^16^, ^18^ During intraoperative evaluation of prolapse, increases in the degree of prolapse have been observed at certain points compared to the preoperative assessment, with differences found in up to 32.1% of patients at specific locations.^15^ This discrepancy is attributed to factors such as patient inhibition of straining or co‐activation of the pelvic floor musculature, elements that are neutralized by the muscle relaxation induced by neuraxial anesthesia, which causes complete paralysis of striated muscles.^17^ This complete relaxation of the pelvic musculature eliminates levator ani co‐activation,^27^ allowing for a maximal and passive exteriorization of the prolapse.

When analyzing the presurgical clinical and ultrasound assessment of multicompartment prolapse, we observed that for cystocele, both tests are effective methods, which is consistent with the literature where the diagnostic agreement between both tests has proven to be very good.^19^ Regarding central compartment prolapse, the POP‐Q system has shown poorer inter‐observer reliability.^8^ We found that presurgical clinical examination was highly sensitive for detecting uterine prolapse but ineffective for identifying cervical elongation, the opposite of which was true for presurgical ultrasound. This dichotomy is of utmost clinical importance, as the differentiation between a true uterine descent and an isolated elongation of the cervix is crucial for surgical planning. We must consider that these differences might be due to how the differential diagnosis is established with each technique. Ultrasound establishes it based on objective measurements,^12^, ^20^ while clinical examination depends on a reference point, limiting itself to describing the anatomy of the vaginal surface.^2^, ^20^

For the posterior compartment, our data reveal an interesting diagnostic dynamic. Clinical examination showed superior sensitivity for the detection of rectocele (61.5%), albeit at the expense of lower specificity (66%). Ultrasound, in contrast, although less sensitive (21.4%), exhibited much higher specificity (86.3%), making it an excellent confirmatory test. Further, presurgical ultrasound demonstrated a far superior ability to diagnose enterocele. This phenomenon could be explained by the fact that clinical examination, by isolating the posterior compartment with a speculum, facilitates the maximum protrusion of the rectocele, whereas ultrasound evaluates the herniation in the context of a global strain,^21^, ^26^ establishing itself as a highly valuable tool for the preoperative detection of often‐occult defects.^20^

### 4.1 Strengths and limitations

The main strength of our study lies in its prospective design, which uses intraoperative evaluation as the reference standard to achieve greater accuracy, along with the strict blinding of evaluators and the use of standardized methodologies that ensure the reproducibility of the findings. Nevertheless, the study is not without limitations. These include its single‐center design, which could affect the generalizability of the results, and a sample size that might be considered small for low‐prevalence defects such as enterocele.

From a clinical perspective, the most direct implication of our findings is that assessment with the POP‐Q system, while fundamental, is insufficient on its own for a complete mapping of the defects. We therefore propose an integrated diagnostic model that routinely incorporates transperineal ultrasound to optimize surgical planning, especially in cases where it is crucial to differentiate the type of uterine prolapse or to detect enteroceles that might go unnoticed clinically. Looking ahead, research should be directed toward conducting multicenter studies to validate these findings and, crucially, toward evaluating whether the implementation of this combined protocol translates into a tangible reduction in long‐term prolapse recurrence rates.

## 5 CONCLUSION

The evaluation of multicompartment prolapse demands a diagnostic approach that integrates multiple sources of information. Clinical examination remains fundamental for quantifying the overall dimension of the descent and demonstrates high reliability in detecting certain apical defects. For its part, transperineal ultrasound proves to be an indispensable tool for precise anatomical characterization, excelling in the differentiation of central prolapse subtypes and in the confirmation of defects such as enterocele. Consequently, the combination of the dimensional assessment provided by clinical examination with the anatomical precision of ultrasound is posited as the optimal standard for comprehensive surgical planning, with the aim of improving patient outcomes.

## AUTHOR CONTRIBUTIONS

Conception and design: José Antonio García‐Mejido. Administrative support: José Antonio García‐Mejido and José Antonio Sainz‐Bueno. Provision of study materials or patients: José Antonio García‐Mejido and Olaya Salas‐Alvarez. Collection and assembly of data: José Antonio García‐Mejido, Fernando Bugatto‐Gonzalez and Olaya Salas‐Alvarez. Data analysis and interpretation: José Antonio García‐Mejido, Ana Fernández‐Palacín and Fernando Fernández‐Palacín. Manuscript writing and Final approval of manuscript: All authors.

## FUNDING INFORMATION

No funding was received for this study.

## CONFLICT OF INTEREST STATEMENT

All authors have completed the ICMJE uniform disclosure form. The authors have no conflicts of interest to declare.

## Data Availability

Research data are not shared.
